# Multiple roles of the coding sequence 5′ end in gene expression regulation

**DOI:** 10.1093/nar/gku1313

**Published:** 2014-12-12

**Authors:** Tamir Tuller, Hadas Zur

**Affiliations:** 1 Department of Biomedical Engineering, the Engineering Faculty, Tel Aviv University, Tel Aviv, Israel; 2 The Sagol School of Neuroscience, Tel Aviv University, Tel Aviv 69978, Israel

## Abstract

The codon composition of the coding sequence's (ORF) 5′ end first few dozen codons is known to be distinct to that of the rest of the ORF. Various explanations for the unusual codon distribution in this region have been proposed in recent years, and include, among others, novel regulatory mechanisms of translation initiation and elongation. However, due to the fact that many overlapping regulatory signals are suggested to be associated with this relatively short region, its research is challenging. Here, we review the currently known signals that appear in this region, the theories related to the way they regulate translation and affect the organismal fitness, and the debates they provoke.

## INTRODUCTION

For many years, researchers referred to the genes’ promoter (which primarily determines the transcription initiation rates) as the main ‘module’ including information regarding gene expression regulation, while the information related to protein structure is contained in the coding sequence via the genetic code. However, various studies have demonstrated that such a modularity is only a raw approximation of the reality. The genetic code is redundant as it includes 61 codons that encode only 20 amino acids; thus, a certain protein can be encoded by a large (exponential) number of codon combinations. Indeed, in recent years, it was demonstrated that multiple ‘overlapping codes’ tend to appear in the coding sequence, related to all stages of gene expression regulation (1–7): for example, replacing a codon with a synonymous one can significantly affect the level of transcript expression. There are some excellent reviews regarding the way information encoded in synonymous codons affects the organismal fitness, resulting in a pattern of non-neutral evolution (8–13). The topic of this review, however, is related only to the 5′ end of the ORF (open reading frame, also referred to as coding sequence or CDS). Specifically, we will discuss only signals that appear in the first 50–70 codons of the ORF.

It has been known for over two decades that the codon frequency distribution at the ORF's 5′ end is different to that observed in the rest of the ORF (14–17). However, only recently have several novel mechanisms been discovered, by which the unusual codon usage bias in this region affects gene translation regulation and organismal fitness.

The current limitations of the experimental approaches for monitoring the regulation of gene translation, added to the fact that this relatively short region is highly occupied with signals related to this process, make the research of this part of the sequence challenging. In the current paper, we review the different signals that appear in this region, the theories related to the way they regulate translation and affect organismal fitness, and the controversies related to some of these signals.

## WEAK mRNA FOLDING AT THE ORF's 5′ END

**Figure 1.**
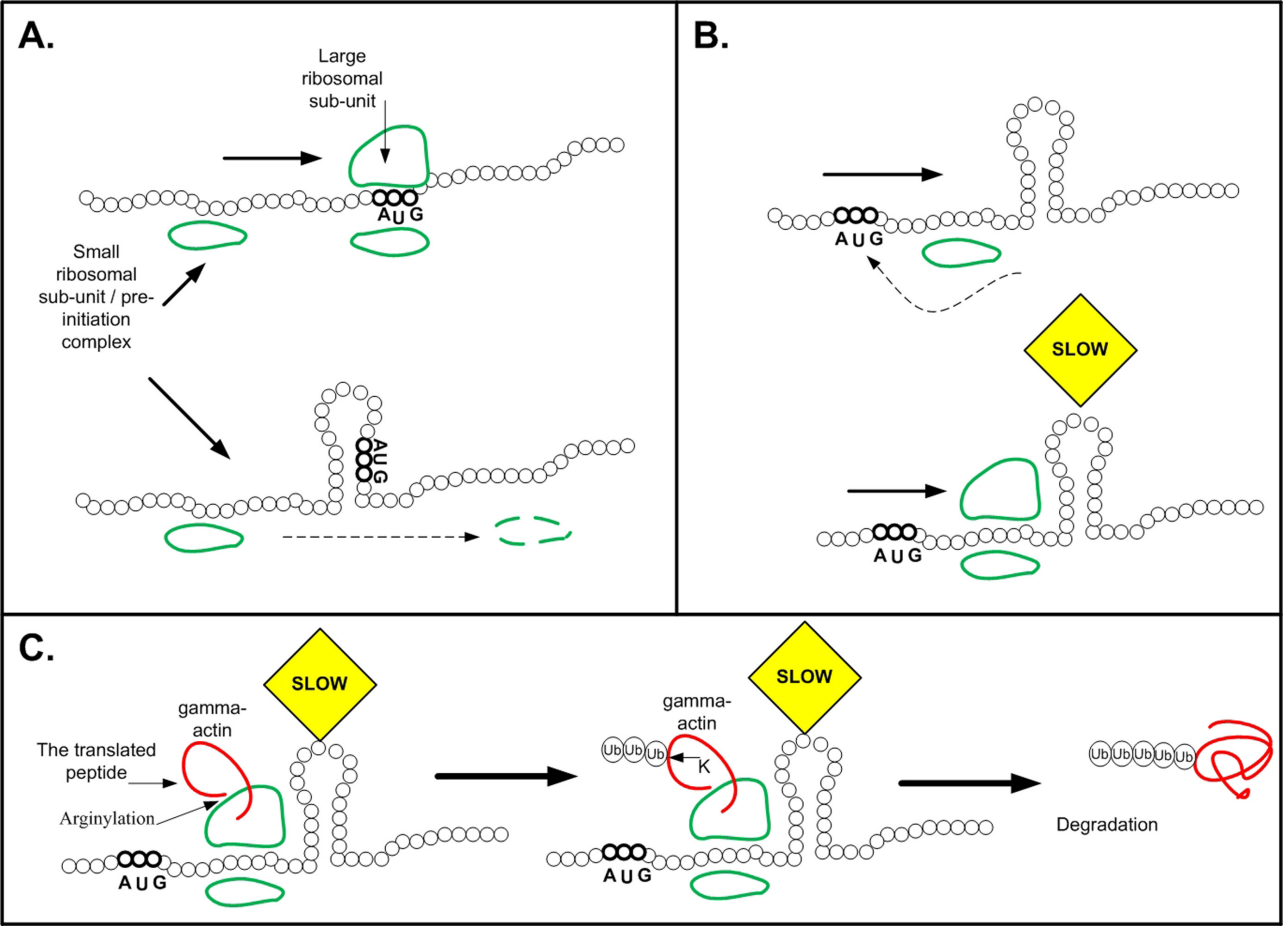
The effect of mRNA folding on translation. All sub-figures include illustrations of the mRNA structure (black) and the ribosomal small and large subunits (green). (**A**) Illustration of how weak mRNA folding at the 5′ end promotes recognition of the start codon by the pre-initiation complex: when the mRNA near the start codon is not folded (top) the pre-initiation complex recognizes the start codon in an efficient manner; when strong mRNA folding surrounds the start codon (bottom) it reduces the affinity of the start codon to the pre-initiation complex. (**B**) Top part: Strong mRNA folding downstream of the 5′ end can improve initiation efficiency by blocking the pre-initiation complex's movement after missing the start codon. Bottom part: Strong mRNA folding downstream of the 5′ end can improve ribosomal allocation and prevent traffic jams by slowing down ribosomes at the beginning of the ORF (and thus increasing the distances between the ribosomes; see illustration in Figure 2A). (**C**) Strong mRNA folding downstream of the 5′ end can also promote degradation of arginylated gamma actin via the exposure of normally hidden lysine residues for ubiquitination (details in the main text).

It was suggested that in the three domains of life bacteria, archaea and eukaryotes, the first 30–40 nucleotides of the ORF undergo evolutionary selection such that there will be weak folding of the mRNA molecule in the region surrounding the start codon (18–24), as the presence of secondary structures inhibits the ability of an mRNA to sequester ribosomes, thereby lowering the effective translation initiation rate. This signal probably promotes efficient recognition of the start codon and the regulatory sequences surrounding it by the pre-initiation complex/30S subunits. If the relevant regulatory sequences, Shine-Dalgarno in prokaryotes (25), and start codon context (usually termed Kozak sequence) in eukaryotes (26,27), and the start codon itself tend to be base-paired to other nucleotides, they do not interact efficiently with the pre-initiation complex/30S subunits (21) (Figure 1A).

Currently, five studies have demonstrated that indeed there is a causal relation between folding at the 5′ end of the ORF and translation efficiency (protein levels) in *Escherichia coli* (18,19,28) and in *Saccharomyces cerevisiae* (29,30). They generated libraries of variants that code the same protein, with each variant being comprised of different synonymous codons (specifically at the beginning of the ORF); and the protein level of each variant was measured. Next, for each variant the folding strength at the 5′ end was computed, and the strength of this folding was found to be negatively correlated with the measured protein levels. It is important to mention, however, that such a correlation was not observed in a sixth study, which analyzed two different reporter genes (31). This shows that the strength of the proposed association between folding at the 5′ end of the ORF and translation efficiency varies among genes, and may even vary among different cellular conditions. Specifically, if factors such as amino acids or tRNA molecules become rate limiting they may blur this relation (see (31) which also analyzed *E. coli*). Finally, it was also observed that in bacteria a high A/U content at the 5′ end of the ORF is correlated with higher protein levels of heterologous protein expression, presumably because high A/U decreases folding (28,32).

## STRONG mRNA FOLDING DOWNSTREAM OF THE ORF's 5′ END

Interestingly, it was also observed in *S. cerevisiae* and some mammals that endogenous genes tend to have *strong* mRNA folding at the region 14–34 codons after the start codon (22,33,34); the fact that this signature is weaker in randomized genomes that maintain the codon bias and protein content of the original genome suggests that this signal is under selection (33). Four explanations may clarify this phenomenon (Figure 1B): First, it was suggested that the strong mRNA folding improves the fidelity of translation initiation by *blocking* the pre-initiation complex scanning, increasing the probability that it will remain in the vicinity of the start codon, and thus increasing the probability that the pre-initiation complex will recognize the correct start codon (22,34–36). Moreover, it was suggested that this signal tends to be selected for when the nucleotide context of the start codon is non-optimal (22,34–36). Second, this signal may be related at least partially to that of the aforementioned *weak* folding at the beginning of the ORF; it is possible the strong structure downstream may help prevent strong folding at the start codon. Third, the strong folding after the 5′ end may delay the ribosomes at the beginning, improving ribosomal allocation and preventing ribosomal collisions and traffic jams (33,37). Finally, it was suggested that strong folding may affect post-translational modification rates and therefore protein levels. For example, mammalian β-actin undergoes arginylation (38), a process in which the enzyme arginyltransferase adds arginine moieties to the protein (39). Surprisingly, the very similar *γ*-actin, is not observed *in vivo* in its amino-terminally arginylated form. The amino acid sequences of the two actin isoforms are highly similar but the RNA coding sequences, specifically codons at their 5′ end, differ. It was found that arginylation of *γ*-actin takes place but is exceedingly unstable, and is regulated as follows: The codons of the γ-actin form a strong mRNA structure that contributes to a slower translation rate of this region (Figure 1C, left), resulting in the exposure of normally hidden lysine residues for ubiquitination; the fact that N-terminal arginylation can attract ubiquitin conjugation machinery (38) (Figure 1C, middle), leads to preferential degradation of γ-actin upon arginylation (Figure 1C, right).

## RELATIVELY WEAK ADAPTATION TO THE tRNA POOl

It was suggested that in both prokaryotes (bacteria and archaea) and eukaryotes the first ∼30–50 codons at the beginning of the ORF tend to be recognized by tRNA species with lower intracellular abundance (6,40,41), resulting in slower ribosomal elongation speed in this region (6,42,43). This region with slower elongation speed and codons less adapted to the tRNA pool was termed ramp (6,40,41), and may provide several physiological benefits.

**Figure 2.**
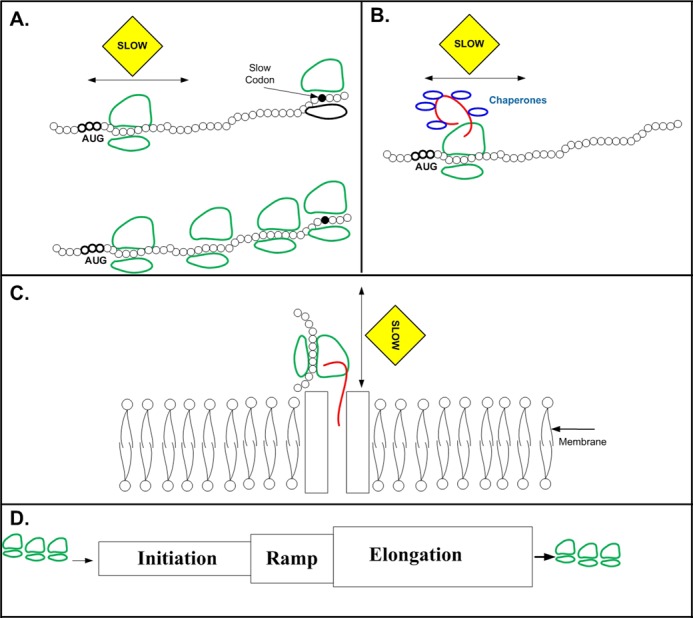
Proposed reasons for the ramp of slower codons, which are less adapted to the tRNA pool, at the beginning of the ORF. The sub-figures include illustrations of the mRNA structure (black), the ribosomal small and large subunits (green), and the translated protein (red). (**A**) Slower codons at the beginning of the ORF improve ribosomal allocation, and prevent jamming and abortion in highly translated genes that have high ribosomal load: Top: When there is a region which is translated at a slower rate at the 5′ end of the ORF it increases ribosome spacing, and decreases the probability of ribosomal jamming and abortion due to slower codons afterward. Bottom: when the region at the 5′ end is not slow, ribosomes have a higher probability to jam and abort downstream from the beginning of the ORF; for example, due to the higher initiation rate there will be a ribosomal jam after a slow codon downstream from the 5′ end (marked in black). (**B**) The slower codons at the 5′ end of the ORF are required for accurate protein folding, and provide sufficient time for chaperon requirement (in blue). (**C**) The slower codons at the 5′ end enable maturation and folding of secretory proteins: the delay caused by the slower codons is required for considering the constraints related to co- and post-translational stages such as membrane translocation and coordinating these stages. (**D**) Illustration: The ramp, region with slower codons at the 5′ end, enables coupling initiation and elongation, and filtering stochasticity due to the fact that it is ‘written’ in the same ‘language’ as the elongation control ‘language’. For example, since the ramp is part of the ORF, a *global* decrease/increase in all the tRNA levels will similarly effect both the ramp and the region downstream of it. Specifically, the *relative* translation rate of the ramp in comparison to the region afterwards will be maintained.

Several explanations have been proposed for the ramp signal (Figure 2): (i) It contributes to increasing the distances between ribosomes, promoting improved ribosomal allocation, and reducing ribosomal collisions and jamming, thus reducing the cost of wasted ribosomes and of spontaneous or collision-induced abortions. (6,8,44). It is important to emphasize that this *ramp was observed mainly in highly expressed genes with high initiation rates and ribosomal density* (6); in these cases, there is a need for traffic control. (ii) It is partially related to assisting maturation and folding of secretory proteins, enabling, among others, co- and post-translational stages such as membrane translocation, protein processing and folding (45,46). (iii) It is known that translation speed can affect co-translational protein folding; thus the ramp of slow codons that are recognized by low-abundance tRNA isoacceptors, may have important contributions to the folding of the first domain of proteins (47–49). (iv) The length of the ramp corresponds remarkably well to the length of the polypeptide needed to fill the exit tunnel of the ribosome (50), so the nascent peptide chain can emerge from the ribosome as it transitions from the slow late-initiation (ramp) stage to the fast stage of elongation. This raises the possibility that the ramp might somehow facilitate interactions between the emerging peptide and the chaperone proteins, thereby increasing the fraction of correctly folded product (40).

Some additional papers supporting the specific importance of the adaptation to the tRNA pool of codons at the beginning of the ORF have been published in recent years. For example, a genome-wide study of natural selection operating on codon adaptation to the tRNA pool in recent human evolution (51). Among others, the analyses suggest that highly expressed genes undergo stronger purifying selection related to the adaptation to the tRNA pool at the 5′ end of the ORF, than in any other part of the ORF (51). Additional studies in *S. cerevisiae* and *E. coli* have demonstrated that the codons at the beginning of the ORF are specifically important for determining translation rates (52,53); expressly, it was suggested that slow ribosome movement near the start codon regulates ribosome recruitment, affecting the initiation rate.

## IS THERE WEAK ADAPTATION TO THE tRNA POOL OR DOES IT ACTUALLY RELATE TO THE SELECTION FOR WEAK FOLDING?

One central debate regarding the signal of weak adaptation to the tRNA pool is related to the fact that it partially overlaps with the signal of weak folding. Thus, it has been suggested that the actual signal that is under direct selection is weak folding, while the observed signal of weak adaptation to the tRNA pool is only a ‘by-product’. This hypothesis was mainly based on synthetic biology experiments in *E. coli* (mentioned above), that include measuring the effect on protein levels based on synonymous perturbations of the codons in this region.

On the other hand, it was shown that in organisms from the three domains of life there is co-evolution between the tRNA pool and the codon distribution of the ORF to maintain this signal (6). Specifically, it was demonstrated that during evolution both codon composition and tRNA levels change; however, the signal of lower adaptation of codons to the tRNA pool at the 5′ end of the ORF is maintained. This evolutionary pattern cannot be explained based on the folding of the 5′ end of the ORF, since mRNA folding is not related to changes in the tRNA pool. Thus, this result suggests that at least part of the observed signal is directly related to adaptation to the tRNA pool.

Moreover, one disadvantage of some synthetic biology experiments is the fact that they may generate sequence variants with extremely strong folding at the 5′ ORF end, in comparison to the weak folding usually observed in this region in endogenous genes (see previous section, (18–24)). Thus, the observed relations in these experiments may not reflect the actual relations in the case of endogenous genes, where folding is weak and adaptation to the tRNA pool becomes rate limiting (54). It is clear based on these experiments, that when randomly perturbing the codons in part of this region (*e.g.* first ∼10 codons) folding has stronger effect on protein levels, probably via its effect on translation initiation. However, it is probable that under the regimen of weak folding that occurs in endogenous genes, the adaptation to the tRNA pool has significant effect on the organismal fitness.

**Figure 3.**
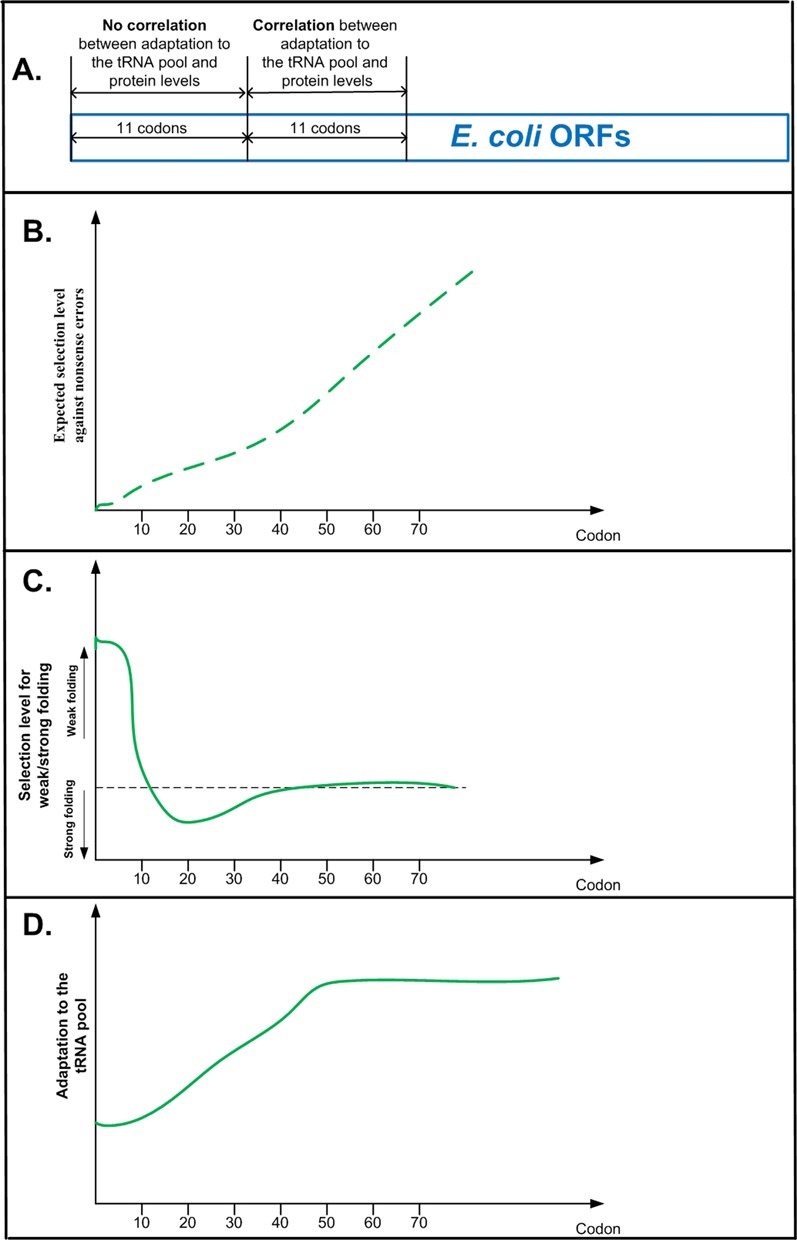
(**A**) Correlation between *local* adaptation of codons to the tRNA pool and protein levels in *E. coli* endogenous genes. When controlling for mRNA folding the correlation is not significant in the case of the first 11 codons, but significant in the case of the second 11 codons (codons 12–22). (**B**–**D**) Comparison of the genomic profiles of selection for adaptation to the tRNA pool and selection for mRNA folding strength, to the expected genomic profile of the cost of nonsense errors. (**B**) Expected profile of selection against nonsense errors: if selection against nonsense errors is the *only* dominant factor shaping codon usage bias along the coding sequence, we expect to see a monotonic increasing level of selection along the coding sequence (as explained in the main text, a nonsense error further away from the 5′ end should cost more than an error closer to the 5′ end of the ORF). (**C**) The profile of selection for weak (strong) mRNA folding: the region under selection for weak folding includes the first ∼10–13 codons; it is followed by a region of selection for strong mRNA folding, and after ∼40 codons there is no specific local signal of selection for mRNA folding (18–24). (**D**) The profile of adaptation to the tRNA pool: the region under selection for weak adaptation to the tRNA pool includes the first ∼30–40 codons (6,40,41).

Finally, most of the synthetic biology studies (for example, (18,32)) researched *E. coli* randomized regions (first 6–11 codons) that are strongly related to folding. Furthermore, some evolutionary studies were also focused only on these regions (32), while the ramp relating to lower adaptation to the tRNA pool is also downstream of those studied randomized regions (it was shown to include the first 30–50 codons (6); see Figure 3). Thus, the outcome of such studies is not informative regarding the effect of lower adaptation to the tRNA pool on organismal fitness in the regions that have not been explored (codons 12–30 in the case of *E. coli* (6)). To demonstrate this point, we computed the partial correlation between protein levels per mRNA (taken from (55)) and the tRNA adaptation index (tAI) (56) given (i.e. when controlling for) the local mRNA folding energy prediction (57) (i.e. r(tAI, protein-levels|folding energy)) in the first 11 codons, and for codons 12–22 in endogenous *E. coli* genes. The correlation was found to be non-significant in the first case (*r* = 0.24, *P* = 0.39 for 16 bins; *r* = 0.04, *P* = 0.25 without binning), but significant in the second case (*r* = 0.60, *P* = 0.017 for 16 bins; *r* = 0.11, *P* = 0.0016 without binning); see illustration in Figure 3A.

## IS THE UNUSUAL CODON USAGE BIAS AT THE ORF 5′ END DUE TO WEAKER INDIRECT SELECTION OR IS IT DIRECTLY SELECTED FOR?

It was suggested that the unusual codon frequency distribution at the 5′ end of the ORF is not due to direct selection related to expression regulation, but due to weaker indirect selection related to codon bias in this region as compared to the rest of the ORF. Specifically, based on the analysis of the *S. cerevisiae* genome it was proposed that this signature is due to weaker selection against nonsense errors during translation (58,59). Since these nonsense errors are expected to cost more when they occur further away from the beginning of the ORF, we expect a stronger level of selection against such errors further away from the ORF 5′ end; as a result, the codon usage bias, which may be partially due to this selection, at the 5′ end of the ORF is weaker (58,59).

On the other hand, while this hypothesis may explain part of the phenomena, previous studies based on non-endogenous/functional genes in *S. cerevisiae* and *E. coli* have demonstrated that the codon bias in this region has direct effect on protein levels (18,19,28–30) and organismal fitness (6,19). Thus, these studies support the conjecture that at least part of the observed codon distribution in this region is directly selected for.

Moreover, the cost of nonsense errors at the beginning of the ORF, which is the sum of the resources invested in producing the erroneous protein, is expected to be proportional to the number of codons/amino acids before the error. Thus, if nonsense errors during translation were the only determinant of codon usage bias, we would expect to see a monotonically increasing profile of selection for codon usage bias (stronger codon usage bias further away from the 5′ end of the ORF). However, the profiles of selection for weak mRNA folding or low adaptation to the tRNA pool have a completely different shape (see Figure 3B–D): the region of selection for weak mRNA folding includes the first ∼10–13 codons, it is followed by a region of selection for strong mRNA folding, and after ∼40 codons there is no specific local signal of selection for mRNA folding (6,21,33). The region with lower adaptation to the tRNA pool is 30–50 codons long, and after this region the adaptation to the tRNA pool is high and there is no specific local signal of selection for the tRNA pool (6).

## WHY IS A REGION WITH SLOWER CODONS NECESSARY IF TRANSLATION CAN BE MODULATED VIA INITIATION RATES?

It is unclear why a single (highly expressed) gene should experience selection both to increase its rate of ribosomal initiation and to reduce the subsequent rate of its early elongation (8). In other words, why do we need a ramp of slower codons if we can modulate translation via initiation (which is assumed to be regulated via the nucleotide context upstream of the ORF).

Various explanations have been proposed for this question (6) (Figure 2): (i) The fact that this region of lower adaptation to the tRNA pool is ‘written’ in the same language (adaptation to the tRNA pool) as the elongation step enables a good coupling between initiation and elongation. Specifically, it was shown that the adaptation to the tRNA pool in this region is not absolutely low, but lower *relatively* to the adaptation downstream of it (highly expressed genes have codons more adapted to the tRNA pool in this region, and even more so downstream of it). (ii) This signal may provide an additional ‘knob’ that can tune down the variance set by the initiation rate on the spacing between ribosomes. (iii) tRNA levels vary among tissues and conditions; thus, this signal, as it is also based on the adaptation to the tRNA pool, enables fitting the initiation to the elongation rate. (iv) Moreover, some of the advantages suggested for this signal (e.g. contribution to folding, chaperon recruitment and protein maturation (40,41,60)), clearly cannot be replaced by initiation regulation. Furthermore, as aforementioned, it was shown that the codons at the beginning of the ORF can actually control the initiation rate (52,53).

## IS THERE AN INCREASED RIBOSOMAL DENSITY AT THE ORF's 5′ END OR IS IT A COMPUTATIONAL/EXPERIMENTAL ARTIFACT?

Assuming a constant flux of ribosomes, higher local ribosomal density is related to lower translation elongation speed (6,19). If indeed the lower adaptation to the tRNA pool at the beginning of the ORF is related to slower ribosomal speed, we would expect an increased local ribosomal density in this region. Thus, a biological phenomenon strongly related to the sequence features of the ORF's 5′ end, is the ribosomal density in this region.

A recent experimental approach named Ribosomal Profiling or Ribo-Seq (43,61) may facilitate answering this question. The method includes the following steps: cells are treated (for example) with cycloheximide to arrest translation, ribosomes are fixed and ribosome-protected RNA fragments (named ‘reads’) are recovered. After processing and reverse-transcription, these are sequenced, mapped, and used to derive ribosomal density profiles. This results with a profile/vector for each coding sequence (named ‘ribosome density profile’ or ‘read count profile’); each position in such a vector is related to one codon, and its value is related to the number of reads (‘read count’) that mapped to that codon. If for a certain coding sequence a ribosome tends to spend more time on codon *x* than on codon *y* (i.e. ‘codon *x* is slower than codon *y’* and the ‘ribosome density in codon *x* is higher than in codon *y’*), the read count related to codon *x* will be higher than the read count for codon *y*.

**Figure 4.**
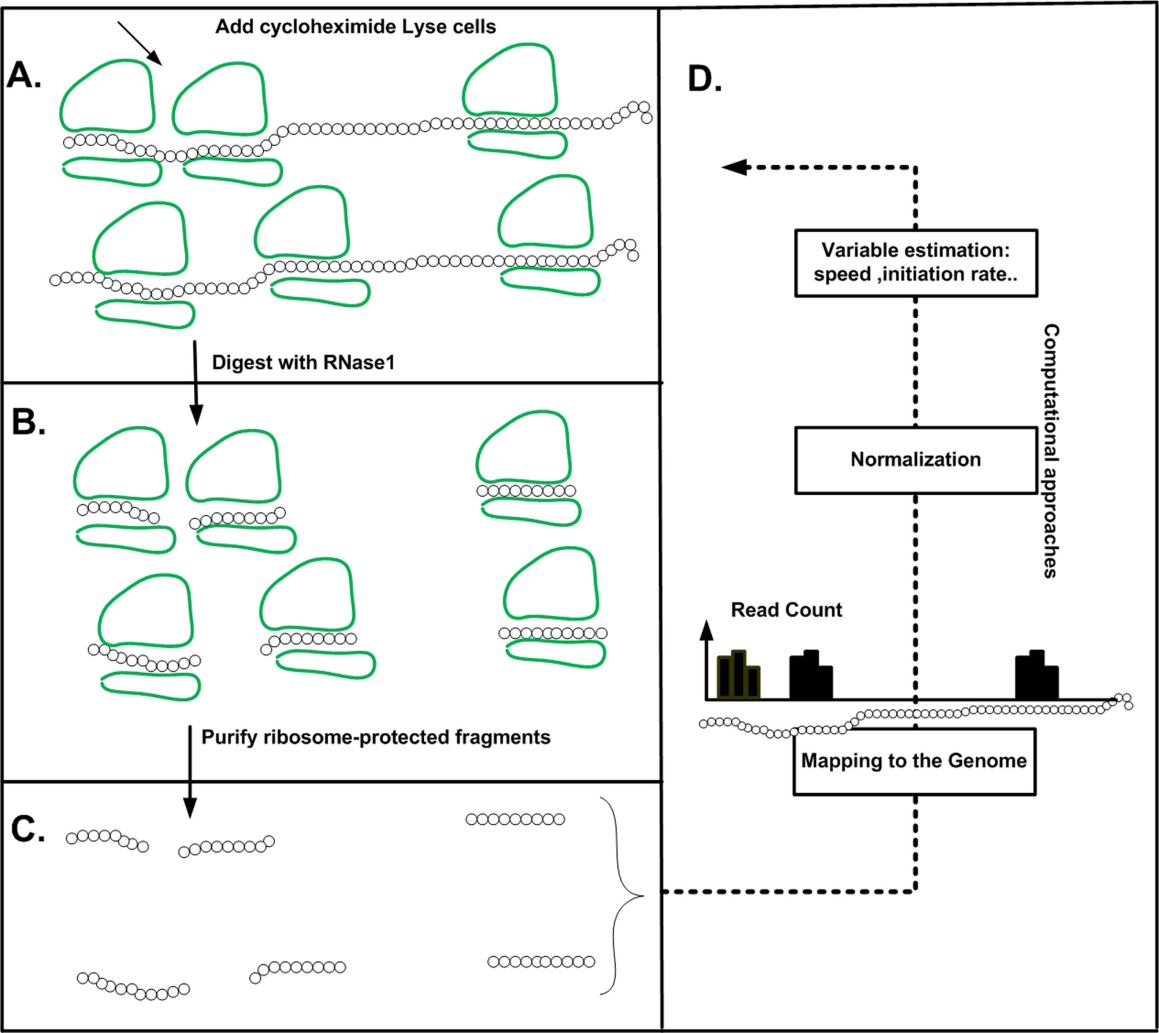
The ribosome profiling approach. The method includes the following steps: cells are treated with cycloheximide to arrest translation (**A**), ribosomes are fixed and mRNA regions not protected by ribosomes are digested (**B**) and ribosome-protected RNA fragments are recovered (**C**). After processing and reverse-transcription, these are sequenced, mapped, and used to derive ribosomal density profiles. The profile includes a vector of read counts for each coding sequence: for each codon (a position in the vector) we have the number of reads that were mapped to this position (**B**). If for a certain coding sequence a ribosome tends to spend more time on codon *x* than on codon *y* (i.e. codon *x* is slower than codon *y*), the read count related to codon *x* will be higher than the read count for codon *y*.

Based on this method, it was found that the region at the beginning of the ORF includes higher density of ribosome-protected RNA fragments, supporting the conjecture that the translation speed is indeed slower in this region (6,43) (see Figure 4). Technically this was achieved by averaging (after some normalizations as described in the following paragraph) the profiles of all the genes to obtain a ‘genomic ribosome density profile’; the position *x* (codon *x*) in the profile includes the mean ‘read count’ when averaging the read count over the position *x* of all the coding sequences’ read profiles.

However, in recent years, there has been an active debate regarding the relation of this signal to a lower translational speed in this region.

First, it was suggested that the higher density of ribosome-protected RNA fragments at the beginning of the ORF is due to experimental biases (62) related to the protocol used in earlier studies (e.g. (43)); these biases increase the read-count mapped to this region (the beginning of the ORF). However, the signal of lower translation speed in this region has been observed also by different experimental protocols and analyses (43,62,63) that should not be affected by the biases that appeared in (43).

**Figure 5.**
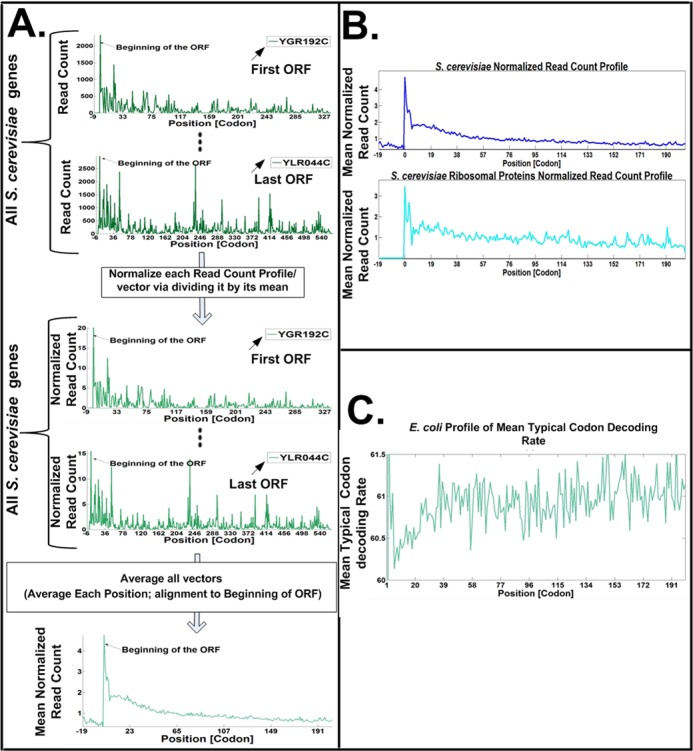
(**A**) The procedure for reporting the mean genomic normalized ribosomal density profile (see, e.g. (64)): Upper sub-figure: the ribosome footprint read-counts (ribosomal density) profile is computed for each gene; each profile includes the number of reads that were mapped for each codon in the gene's ORF (see also Figure 4). Middle sub-figure: each individual gene's ribosomal density profile is normalized by dividing by its mean read count; at this stage, the mean read count of each of the gene profiles is identical. Lower sub-figure: the normalized read count profiles are aligned to the start codon; a mean genomic read-count profile is obtained by averaging all the individual profiles (for each codon we compute the mean normalized read count for this codon across all the genes). Under this procedure each gene ‘contributes’ similarly to the mean profile; thus, there should not be an increased ribosomal density at the 5′ end of the ORF, if ribosomal density is only due to higher initiation rates (and thus higher read counts and ribosome density) in shorter genes. The fact that the mean *normalized* read count profile of local ribosomal density is higher at the 5′ end (as can be seen in the figure) demonstrates that higher initiation rates in shorter genes can't explain this phenomena. (**B**) Mean normalized read count profile of local ribosomal density for the entire gene set and for the ribosomal proteins of *S. cerevisiae*. In both cases the normalized ribosomal density is higher at the 5′ end. (**C**) Mean genomic profile of the typical codon decoding rates in *E. coli*: the typical decoding rate of each codon is obtained based on a novel statistical filter that controls for phenomena such as experimental biases (specifically at the 5′ end of the ORF), extreme (but possibly rare) ribosome pauses, and ribosome traffic jams. The profile suggests that the first 30–40 codons in *E. coli* indeed tend to include codons with lower nominal/typical translation rates.

Moreover, recently a new approach for estimating the nominal elongation speed of codons was suggested (64). Specifically, this approach filters the biases and extreme values (e.g. ribosome pauses) that appear in Ribo-Seq (expressly the biases at the beginning of the ORF), and considers other phenomena such as ribosome traffic jams (64). When the genomic mean ribosomal speed was computed based on this approach the elongation speed was still lower at the beginning of the ORF (Figure 5).

It was also shown, based on Ribo-Seq analysis, that wobble interactions slow down the ribosome (65). This study also supports the fact that lower adaptation to the tRNA pool in this region indeed affects ribosomal speed. When analyzing Ribo-Seq data, it is important to remember that probably additional biases related to the Ribo-Seq protocol are yet to be discovered and understood (66).

Second, based on the analysis of *S. cerevisiae*, it was suggested that the observed signal of higher ribosomal density at the 5′ end is an artificial result related to the fact that shorter genes have higher initiation rates: higher translation initiation rate should increase the number of ribosomes on the ORF (i.e. ribosomal density) (29); thus, in the genomic ribosomal density profiles where the mean ribosome density for each codon is computed over the entire set of genes (6,43), the short genes (with high ribosome density) contribute only to the first codons, and eventually increase the mean ribosomal density in these codons. However, since the adaptation to the tRNA pool and codon usage bias are lower at the beginning of the ORF (6), this claim (29) actually dismisses the more fundamental observation that lower codon usage bias (or adaptation to the tRNA pool) corresponds to higher ribosomal density (see, e.g. (19)).

Moreover, the previous studies that reported a genomic profile of increased ribosomal density at the beginning of the ORF were based on *normalized* ribosomal density profiles (6,33,43). Specifically, the ribosomal density profile of each ORF is normalized by dividing all the read counts of each profile position by the mean read count of the profile (and thus each ORF contributes in a *similar* way to the genomic ribosome density profile, regardless of what its initiation rate and mean ribosome density are). It was shown that in this genomic normalized ribosome density profile there is still increased ribosomal density at the 5′ end of the ORF (Figure 5). If the observed profile was only due to the higher read counts of shorter genes the normalized genomic profile of *S. cerevisiae* should have been flat.

In addition to the aforementioned points, when considering only the ribosomal proteins, which have relatively similar length and expression levels, the increased ribosomal density at the 5′ end of the ORF can still be observed (Figure 5).

Furthermore, the analysis performed in (29) considered all genes, while the analysis in (6,33,43) considered only highly expressed genes. The ribosomal profiling approach provides a very limited cover for non-highly expressed genes: when *not* considering 20% of the genes with top ribosomal footprint density, <7% of the positions of the remaining genes are mapped to ribosome-protected RNA fragments in *S. cerevisiae*. Thus, analyzing other groups of genes (i.e. not highly expressed) is unreliable. Moreover, as aforementioned, the signal of lower adaptation to the tRNA pool is expected to be observed mainly in highly expressed genes (6). Finally, the proposition above cannot explain results found via other/similar approaches; for example, as mentioned above, a novel approach was suggested for estimating the nominal/typical decoding rates of codons while filtering phenomena such as experimental biases, extreme (but rare) ribosomal pauses and ribosome traffic jams (64). Analyses based on this approach still suggest slower translation elongation rates at the beginning of the ORF (Figure 5C).

## INTERACTION WITH THE PRE-INITIATION COMPLEX IN EUKARYOTES

In prokaryotes translation initiation is known to be mediated via the Shine-Dalgarno (SD) sequence (25), a ribosomal binding site in prokaryotic mRNA, generally located around eight bases upstream of the start codon AUG. The canonical eukaryotic translation initiation model includes scanning of the transcript from the 5′ end toward the 3′ end until a start codon is recognized. Thus, it has been established for many years that the immediate nucleotide composition surrounding the start codon can affect the translation initiation rate and fidelity via its interaction with the pre-initiation complex (26,27). Specifically, based on the analyses of various eukaryotes, it was suggested that the first 3 nucleotides of the ORF following the start codon have a major effect related to this signal (26,27,35,67–69).

Later studies have demonstrated that there are additional signals related to the interaction with the pre-initiation complex that are encoded at the beginning of the ORF. For example, based on the analysis of 33 eukaryotes, it was shown that there is selection for fewer start codons in all frame shifts in the first 5–11 codons of the ORF (35). In addition, it was shown that in all frames there is selection for ATG codons with anti-optimal contexts in the first few dozen codons of the ORF (35). Recent studies in various eukaryotes have demonstrated that ATG (AUG) codons in the vicinity and downstream of the main start codon of the ORF can indeed trigger initiation events (70–76). Thus, these experimental results support the conjecture that ATG codons, and optimal context scores of ATG codons, are selected against in this region to prevent undesired translation initiation events.

## INTERACTION OF THE AMINO ACID COMPOSITION AT THE N-TERMINUS WITH THE RIBOSOME

It is known that the frequency of different amino acids (amino acid bias) is different at the ORF 5′ end than afterwards. For example, many proteins include a signal peptide, which is a short (5-30 amino acids long) peptide present at the N-terminus of the proteins that are destined towards the secretory pathway (77).

During protein synthesis, nascent peptides leave the ribosome through the ribosomal exit tunnel. Thus, because of the unique biochemical properties of the exit tunnel, both in eukaryotes and in bacteria (and probably also in archaea) specific short peptides may undergo strong biochemical interactions with the exit tunnel (78–81). For example, based on the analysis of ribosomal profiling data, two recent studies have demonstrated that in *S. cerevisiae* positively charged amino acids of the growing translated peptide tend to interact with the negatively charged exit tunnel of the ribosome and slow it down (33,82).

When dealing with amino acid bias, it is hard to prove selection related to translation: changes in amino acid content may affect translation via the interaction with the ribosomal exit tunnel (for example), but can clearly also affect the functionality of the protein. It was demonstrated that proteins in *E. coli* and in *S. cerevisiae* tend to include positively charged amino acids at their 5′ end (33). Specifically, it was shown that both in *E. coli* and in *S. cerevisiae* proteins from all cellular functions tend to have higher frequencies of positive amino acids at their 5′ end. Thus, it is plausible to speculate that this signal may be at least partially related to selection for decreasing elongation speed in this region due to reasons mentioned above (in the sections related to mRNA folding and adaptation to the tRNA pool signals).

However, this result is also under debate. It was suggested that this signal is only due to membrane proteins that tend to have positively charged amino acids in this region (83). Nonetheless, it was shown that this signal is not due to specific proteins (for example, membrane proteins or heat shock proteins), and appears in highly translated proteins that are not membrane proteins such as ribosomal proteins (33).

**Figure 6.**
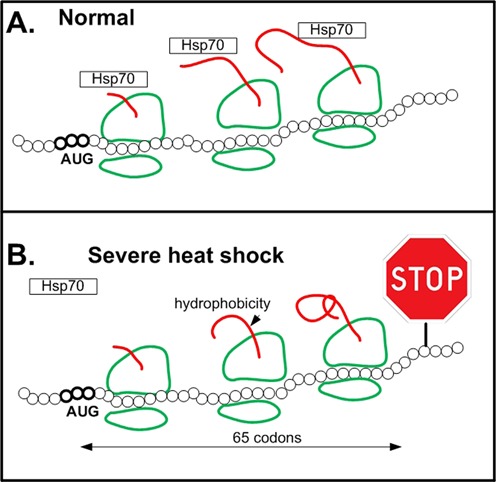
An illustration of how severe heat stress triggers global pausing of translation elongation in the vicinity of codon 65 in proteins with hydrophobic N-terminus via down regulation of Hsp70. (**A**) In normal conditions Hsp70 contributes to accurate protein folding during translation elongation. (**B**) The N-terminus of proteins tends to have hydrophobic amino acids that cause (among others) miss-folding/aggregation, and can interact with the ribosome exit tunnel. During severe heat stress chaperons such as Hsp70, that normally prevent miss-folding and aggregation, are down regulated; thus, these problems result in ribosomal pauses on average after 65 codons.

Irrespectively of this debate, an additional translational mechanism which is mediated by the amino acid content at the N-terminus of the protein has been recently suggested (84) (Figure 6). Studying Mouse fibroblast 3T3 cells Shalgi *et al.* have shown that 2 hours of severe heat stress triggers global pausing of translation elongation in the vicinity of codon 65. This phenomenon is related to the fact that the N-terminus of proteins tends to have hydrophobic amino acids that cause (among others) miss-folding/aggregation, and can interact with the ribosome exit tunnel. During severe heat stress chaperons such as Hsp70, that normally prevent miss-folding and aggregation, are down regulated. Thus, these problems result in ribosomal pauses on average after 65 codons. It is not clear (and not reported in (84)) why (on average) the ribosomes tend to pause after 65 codons. However, according to (84), it is probably a function of the following parameters: the function and binding sites of HSP70; the geometry and length of the ribosome exit tunnel (exit tunnel length is around 31 codons (78)); the distribution of hydrophobic amino acids at the 5′ end of the ORF (according to (84)), there is a peak/ramp of hydrophobic amino acids at the first ∼20–25 AAs/codons); the type of the possible interactions between HSP70 and the ribosomal exit tunnel (84).

## ARE CHARGED RESIDUES THE MAJOR DETERMINANTS OF TRANSLATION RATE OR DO OTHER FEATURES OF THE TRANSCRIPT SIGNIFICANTLY CONTRIBUTE?

Recently, based on the analysis of Ribo-Seq data in *S. cerevisiae* (data was downloaded from (43)), it was suggested that the positions of positively charged residues are the major determinants of translation rates (82). Specifically, they found that on average the mean read count downstream of a positively charged amino acid(s) is higher than in other regions. In addition, they did not find an increase in read count near rare codons or regions with strong mRNA folding (85).

However, while the tests above support the conjecture that positively charged amino acids slow ribosomal elongation speed (as was suggested in other studies (33,63,78)); the analysis (and thus the conclusions) regarding the (lack of) effect of the adaptation to the tRNA pool and mRNA folding on ribosomal elongation speed is wrong. Specifically, (82) studied only one organism (*S. cerevisiae*) and one ribosome profiling data (43), thus the conclusions are not universal; these data were later reported to have biases (see, e.g. (62,66)), but these biases were not considered in (82), and probably contributed artificially to the reported signal. For example, if the Ribo-Seq reads tend to be enriched with adenosine (‘A’), and since positively charged amino acids such as ‘Lys’ tend to be ‘A’ rich (82), it may artificially contribute to the relation reported in (82).

Indeed, papers that were published both before and after (82) have demonstrated that together the mRNA folding, adaptation to the tRNA pool, and amino acid charge significantly contribute to elongation speed:

First, the data analyzed in (82) were also analyzed in a different study (33) where it was found that elongation is determined by amino acid charge, mRNA folding, and adaptation to the tRNA pool. Another study in a different organism (mouse), and based on a different and improved Ribo-Seq protocol (63) has shown that in this organism the three variables (amino acid charge, mRNA folding and adaptation to the tRNA pool) contribute to elongation speed.

Furthermore, a recent study (86) has shown that mRNA folding has an important contribution to elongation speed, while additional recent studies (64,87) have shown that adaptation to the tRNA pool correlates with translation elongation both in eukaryotes and prokaryotes.

Finally, as aforementioned, analyses of heterologous gene expression have shown that mRNA folding, and adaptation of codons to the tRNA pool, significantly affect protein levels and organismal fitness both in eukaryotes and prokaryotes (see, e.g. (19,30)).

## THE EFFECT OF mRNA FOLDING AT THE ORF 5′ END AND mRNA DEGRADATION RATE

In some bacteria mRNA degradation is mediated, amongst others, via 5′ exonucleases (88,89). Thus, it is natural to speculate that features of the 5′ end of the ORF encoded via the codon distribution affect the efficiency of this process. Indeed, previous studies found a negative relation between mRNA folding strength, specifically in this region, and mRNA half-life in *E. coli* (19,90,91). In addition, a negative relation between GC content, specifically in this region, and the degradation rate was observed in *E. coli* (19,90). However, a similar relation was not observed in eukaryotes such as *S. cerevisiae* (92). It was suggested that this relation is related to the translation step: weak folding or AU rich sequences in this region improve translation initiation and thus ribosomal density; moreover, ribosomes protect the mRNA molecule from being degraded.

## DISCUSSION

**Figure 7.**
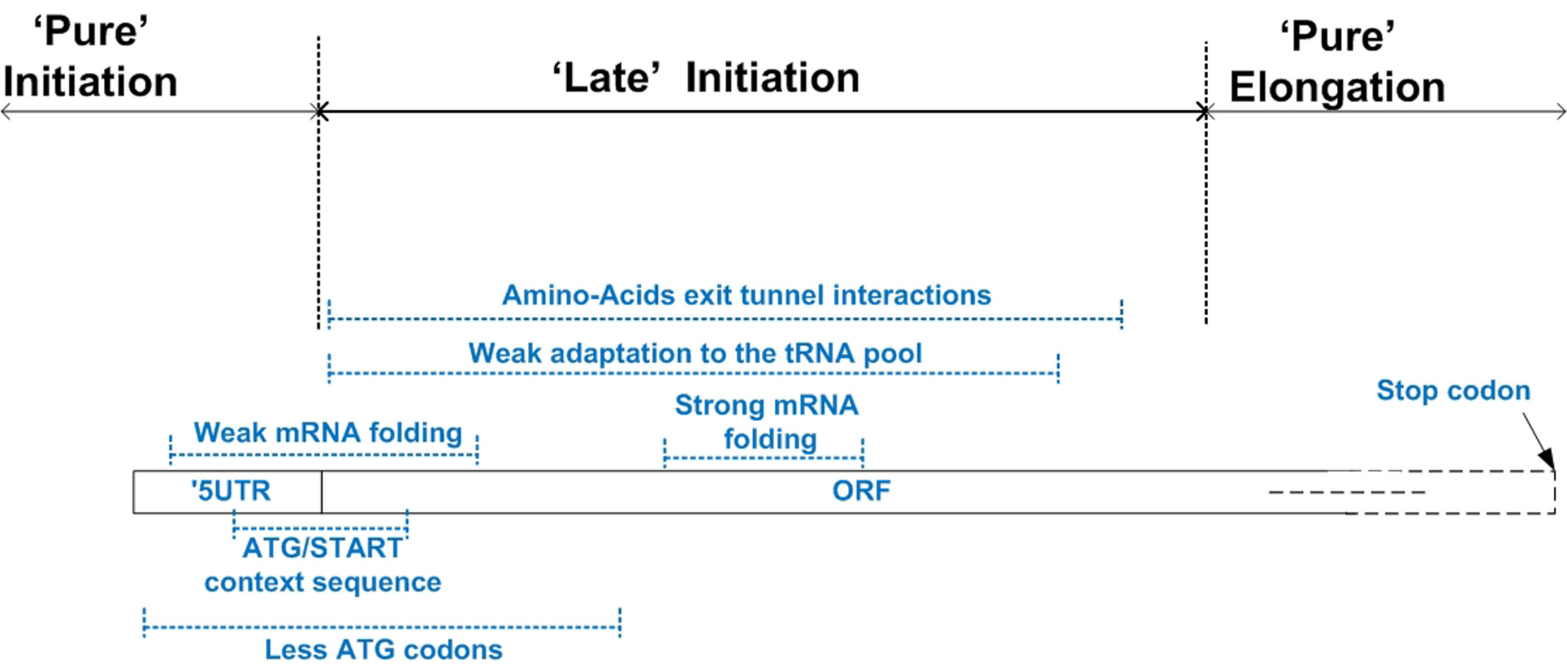
Summary of the reported regulatory signals encoded at the 5′ end of the ORF. The Figure zooms into the first 70 codons of the ORF; it includes the region in the transcript where each signal tends to appear (dotted blue lines with the corresponding signal near the line). The figure emphasizes the beginning of the ORF as a late initiation region, which couples the initiation and elongation steps; this, region is part of the coding sequence but also includes signals that regulate the initiation step.

In this paper, we report the various known translation regulatory signals appearing at the 5′ end of the ORF. We showed that this region is highly populated with patterns related mainly to the initiation and the elongation steps of translation, but also to other aspects and stages of gene expression. The signals are encoded via various properties of the mRNA sequence (e.g. folding), its interaction with intracellular molecules (e.g. tRNAs and ribosomes), and the protein it encodes (properties of amino acids such as charge and hydrophobicity); see Figure 7. Some of these signals have already proved to be universal as they appear in organisms from the three domains of life, however, most of them are either specific to some organisms, or yet to be studied in additional domains of life.

It has previously been suggested that the effect of the reported signals on organismal fitness and the biophysics of translation may induce additional signatures at the beginning of the ORF. For example, it was shown that the beginning of the ORF exhibits increased robustness to transcription errors in terms of their effect on folding energy (33). Furthermore, it was suggested that slower codons tend to have higher translation error (93,94); thus, there should be an increased translation error rate at the beginning of the ORF due to lower adaptation to the tRNA pool, and slower elongation speed in this region (6,63).

The specific importance of the first codons of the ORF is related to the fact that they are at the interface between the 5′ UTR (where signals corresponding to the initiation step are encoded), and the ORF (where the protein and signals related to the elongation step are encoded). Thus, this region serves as a late initiation region that couples the initiation and elongation steps; see Figure 7.

The uniqueness of this region of the transcript may suggest that the evolutionary selection on synonymous and non-synonymous mutations occurring in it is different than in other regions (6,8,21,95). Thus, understanding the effect of the nucleotide distribution in this region should promote developing novel and more accurate models of transcript evolution; such models should consider the effect of mutations in this region on the organismal fitness based on their effect on gene expression. It may also suggest that mutations in this region tend to have an elevated effect on human health, and contribute to the pathogenesis of various diseases (10). In addition, accurate engineering of this region is important for developing novel approaches for efficient heterologous gene expression, and for promoting other biotechnological objectives (18,96). Furthermore, as depicted in this review, the study of this region should have important contributions in the field of functional genomics. Thus, a more advanced knowledge of the coding region's 5′ end is expected to contribute to all biomedical disciplines.

It was suggested that in (specifically) higher eukaryotes (with small effective population size and huge genomes) we should not expect to explain all aspects of gene structure in terms of natural selection (see, for example, (97)). However, it is important to mention that all the signals that appear in Figure 7 were reported also in organisms such as bacteria and/or fungi; these species are known to have very large effective populations and strong selection for codon usage (97–99). This fact supports the conjecture that these signals are under selection in at least some of the organisms. Moreover, as aforementioned, these signals were shown to experimentally regulate gene expression (thus, even if they are not under selection they are clearly important from the functional genomics point of view).

The fact that so many regulatory signals are populated in this relatively short region makes its research especially challenging. Here, we have surveyed some of the current debates related to the nature of the regulatory signals encoded in it. We would like to emphasize that we usually believe that the multiple theories, or relevant variables, related to this region co-exist (54). A good analogy to the stochastic aspects of the evolutionary process is the ‘tinkerer's work’—a ‘tinkerer’ who does not know exactly what he is going to produce but uses whatever he finds around him whether it be pieces of string, fragments of wood, or old cardboards (100–102); this means that evolution is *not* like an engineer that usually searches for modular, easy to manipulate/analyze/maintain, solutions; thus, often searching for a single simple explanation for a biological phenomenon may be wrong. When studying the transcriptome of a certain organism it is important to remember that it is shaped by various environmental conditions, and that it is a superposition of many individual gene groups. For example, the selection for unusual codons in this region is related both to translation regulation via mRNA folding, and via adaptation to the tRNA pool; ribosomal density in this region is expected to be higher specifically in tightly regulated highly expressed genes, while in lowly expressed genes such a signal may be very weak or not exist at all; both translation regulation and protein function shape the amino acid content in this region at the genomic level; elevated ribosome density at the beginning of the ORF may be related both to experimental biases and to slower translation rates in this region.

One important aspect related to this topic is the fact that with conventional statistical approaches one cannot clearly provide confidence levels related to negative results (lack of relation); while *P*-values are conventionally used for reporting the existence of statistical relations between variables, it is not clear how to deal with proving lack of relation (with such relations being reported in previous studies). Many studies aim to ‘prove’ that only one theory or variable is relevant, while not using the best/accurate measures/approaches for evaluating the additional (not mutually exclusive) theories/variables. In these cases, it is usually possible to only accept the positive results but not the negative ones. We believe that more than 1-3 different variables are necessary to explain the effect of the nucleotide distribution at the ORF 5′ end on the organismal fitness and gene expression (while in most previous studies only 1-3 different variables are discussed). Specifically, we believe that, for example, inferring a model that explains the organismal fitness based on 10 sequence variables (for example, each has a correlation of ∼ 0.32 with organismal fitness) is not trivial at all given the following reasons: (i) there are (at least) thousands of potential sequence features that may be relevant in this context (see, e.g. (103)); (ii) the various gene expression steps include many sub-steps that involve dozens of relevant proteins; (iii) gene expression is condition dependent, while the transcript features are ‘static’; (iv) from the evolutionary point of view a few percentages of difference in the fitness is very significant (an allele that improves the fitness by 4% is expected to take over the entire population after several dozen generations).

Furthermore, it is challenging to directly estimate the effect of the different signals on the organismal fitness. To the best of our knowledge, only two studies (19) provided such an estimation in *E. coli* based on the analyses of heterologous gene expression: in the first (19) it was suggested that the effect of lower adaptation to the tRNA pool may explain up to ∼30% of the variance in organismal fitness (measured in growth rate). Based on these data, another study estimated that the region with weak adaptation to the tRNA pool at the beginning of the ORF can explain up to ∼36% of the variance in organismal fitness (measured in growth rate) (6).

The ability to estimate the effect of complex features of the transcript on the organismal fitness is clearly a very challenging endeavor, which is currently impossible due to the following reasons:

(1) Many of the features can potentially have strong effect on fitness; however, it is impossible to deduce this effect based on the analysis of endogenous genes and their expression levels, the data used in almost all the papers on this topic:

First, frequently (all) endogenous genes undergo selection to improve a certain feature in a relatively uniform manner. Since in all genes the feature is ‘close to optimal’, there is very low correlation between the value of this feature and measurements of gene expression, while in practice such a feature can have a very strong effect on gene expression and organismal fitness. For example, via the analysis of heterologous genes and their expression in *E. coli* it was shown that the folding strength near the beginning of the ORF strongly correlates with protein levels (*r* = 0.66 (19)); however, when performing a similar analysis based on endogenous genes there is no correlation (*r* = 0.019 (20)). Based on this example, we can conclude that the folding at the beginning of the ORF has strong effect on gene expression, but this cannot be deduced via the correlation of this feature with gene expression in endogenous genes. Second, often a certain feature is extremely important for a certain set of genes (e.g. highly expressed genes, or genes with a certain function), and not very relevant for other types of genes; in such cases, the feature can be important, but simple correlation of this feature with gene expression cannot infer its effect on fitness. For example, the mechanisms described in Figure 1C and in Figure 6 are clearly important, but relevant only to some of the genes.

(2) Almost all the heterologous gene expression studies on this topic are based on non-functional heterologous protein levels (that do not interact or/and regulate the host pathways) of only one gene. However, to accurately estimate the effect of various features on organismal fitness it is not enough to study one gene in one condition, and it does not suffice to measure only protein levels:

First, the effect of a certain variable on organismal fitness depends on the value of other variables; thus, a heterologous gene expression study of one gene can be very misleading. For example, if the analyzed variants of the heterologous gene have strong mRNA folding at the 5′ end, this can blur the relation between expression levels and adaptation to the tRNA pool, since the folding becomes the rate-limiting variable. However, in endogenous genes there is selection for weak folding of the mRNA in this region, and adaptation to the tRNA pool is rate limiting (see, for instance, (20,54,104), for more details regarding this example).

Second, measurements of protein levels are insufficient for evaluating the effect of transcript features on fitness; often additional measurements such as growth rate and/or other relevant variables are required. For example, in (19), the strong effect of adaptation of codons to the tRNA pool was observed only when correlating measurements of codon usage bias with growth rate; however, low correlation with protein levels was obtained.

Third, as mentioned many of the signals should affect fitness only when they appear in specific gene groups (e.g. genes with high expression levels), and/or are relevant in specific conditions. For example, it was suggested that slower codons at the 5′ end of the ORF improve ribosome allocation and prevent collisions (6); however, trivially we expect to see the affect of this signal on fitness only in the case of genes that consume many ribosomes (if a transcript does not occupy many ribosomes there is no need to optimize their trafficking). Thus, if the analyzed (non-functional) heterologous gene is not highly enough expressed we may simply not see any effect on fitness.

Fourth, many of the signals reported in this review are related to the function of the protein in a non-trivial way (e.g. Figure 2B and C); since almost all heterologous gene expression studies are based on ‘non-functional’ genes from the host ‘point of view’ (e.g. GFP protein) they can't evaluate these signals. However, the study of heterologous expression of functional genes is clearly more complicated than the study of non-functional genes, due to their effect and interactions with the endogenous genes of the host.

(3) Some of the signals reported here are based on a genomic average and are distributed over the entire transcriptome; it is possible that to generate perturbations with a detectable effect on the organismal fitness *many* endogenous genes need to be manipulated. It is feasible to study this topic via analysis of heterologous genes, but such a study may not reflect the endogenous regimen.

To be able to estimate the relative effect of different coding sequence determinants on a certain organism's fitness one should perform heterologous gene expression experiments that include: (i) many genes in (ii) various expression levels; (iii) in addition to protein levels they should include measurements of growth rate and possibly additional gene expression variables (e.g. ribosome densities and RNA polymerase densities); (iv) furthermore, the analyzed heterologous genes should be functional. This is clearly a very challenging project that will hopefully be the topic of future studies.

Finally, in this paper, we focus on signals related to organisms from the three domains of life; most of the signals were reported in eukaryotes and bacteria, but some of them were also observed in archaea. We would like to reemphasize the fact that many of the reported signals have been observed in only one or two model organisms (usually model organisms such as *E. coli* and *S. cerevisiae*). Various papers (e.g. (105,106)) have reported that codon usage bias varies among bacteria, from strong in some species (like *E. coli*), to weak or non-existent in others. Thus, it is very probable that the signals reported here are not common to all the domains of life and/or different subgroups within each domain. Specifically, we believe that the strength of at least some of these signals will be weaker in organisms with smaller effective population size, which tend to have lower levels of selection for codon usage bias (98). Mammals are an example of a group of organisms with small effective population size. Numerous papers have reported that codon usage bias in mammals is influenced by the ‘isochore’ structure of the genome (G+C-content variation), and not by selection for translation (107,108) (note however, there are studies that were able to connect this bias also to translation (51,109)). Nevertheless interestingly, as aforementioned, most of the reported signals were observed also in some mammals. It is not clear if the reported signals occur in other mammals or other eukaryotes, archaea and bacteria. This topic should clearly be further explored in the future.

We want to emphasize that viral genes are also extremely populated with gene expression signals at their 5′ end. For example, the beginning of the Dengue virus and the family *Picornaviridae's* ORFs include several functional mRNA structures (110,111); and dsDNA viruses exhibit selection for reduced stability of mRNA secondary structure near the translation-initiation site (112).
